# In island containment: a qualitative exploration of social support systems among asylum seekers in a mental health care programme on Lesvos Island, Greece

**DOI:** 10.1186/s13031-019-0218-9

**Published:** 2019-07-22

**Authors:** Maria Episkopou, Emilie Venables, Katherine Whitehouse, Christos Eleftherakos, Federica Zamatto, Francisco de Bartolome Gisbert, Nathalie Severy, Declan Barry, Rafael Van den Bergh

**Affiliations:** 1 Médecins Sans Frontières-Operational Centre Brussels, 118 Aristidou, 17672 Athens, Greece; 2 Médecins Sans Frontières-Operational Centre Brussels, Luxembourg Operational Research Unit (LuxOR), Luxembourg, Luxembourg; 3 Division of Social and Behavioural Sciences, School of Public Health and Family Medicine, University of Cape Town, Cape Town, South Africa; 4 Operational Department, Médecins Sans Frontières-Operational Centre Brussels, Brussels, Belgium; 5 Medical Department, Médecins Sans Frontières-Operational Centre Brussels, Rome, Italy; 6 Medical Department, Médecins Sans Frontières-Operational Centre Brussels, Brussels, Belgium

**Keywords:** Social support, Qualitative research, Operational research, Migrants, Asylum seekers, Containment, Isolation

## Abstract

**Background:**

Social support is a core determinant of health and plays a key role in the healing process of people with mental health problems and those who have been exposed to torture or other traumatic events. At the same time, social support is particularly challenging to build in such populations, as self-isolation and social withdrawal are common consequences of traumatic incidents. Defining social support is also challenging as there is no globally adequate definition. Our aim was to explore how social support was understood by Médecins Sans Frontières (MSF) beneficiaries, and how they perceived their needs on Lesvos Island, Greece to be met.

**Methods:**

This was a qualitative study, based on exploratory free-listing interviews that explored how MSF beneficiaries on Lesvos understood and defined social support, followed by a series of in-depth interviews through which participants explained how they perceived their needs to be met. The study was conducted over a period of two weeks in August 2018, with 32 migrants and asylum seekers (22 male, 10 female) enrolled in the mental health services of MSF on Lesvos Island. The majority of interviewees were single men of African origin who had resided in Moria camp between 2 months and 2.5 years. Countries of origin include Syria, Afghanistan, Cameroon, Democratic Republic of Congo (DRC), Iraq, Iran, Nigeria, Senegal and other West African countries.

**Results:**

Participants defined social support as the practical, informational and emotional support that people receive from organisations, friends and family members. Results revealed a lack of community links, isolation, tensions and conflict, insufficient amenities and limited orientation to services that lead to and amplify isolation, discrimination and tension. Most of the participants received little or no support both formally from organisations and informally from other migrants and asylum seekers in the camp.

**Conclusions:**

Functional support networks are urgently required to overcome the consequences of restrictive policies which force people into containment and remove their support systems. Actors who are involved in providing social support, including MSF, are strongly encouraged to engage in activities that work towards building and strengthening peer support networks and creating a sense of community.

## Background

Social support and social networks are core determinants of health [1], and can be defined in a variety of ways [2–4]. These definitions are heterogeneous and scholars argue that even existing definitions can be inadequate because social support is comprised of multiple overlapping concepts and has to be contextualised [5]. For the operational purposes of MSF’s work, social support is defined through two main aspects: functional support and structural support [6, 7].

Functional support refers to the provision of specific forms of support including practical, informational, emotional, financial and what is referred to as meaning-making, whereas structural support refers to the members of the person’s social network. Providers of social support can include informal networks of family and friends as well as civil society actors and governmental service providers. The number of people, social ties and the frequency of interaction play an important role in the overall impact that social support can have on people’s lives [4, 8].

Having social support can empower individuals to use their own resources to cope with difficulties, and a lack of such support can have detrimental consequences, especially during major life transitions such as migration and resettlement [4, 9–12]. As such, we interpret social support in the framework of the coping theory of Stewart [13], where social support processes represent coping resources. Social support is particularly important for the mitigation of major stressors such as poor living conditions in camps or shelters, unemployment, and the loss of previous social networks during and post-migration [14, 15].

Social support is one of the main determinants of resilience and plays a key role in the healing process of migrating populations who have been exposed to torture or other traumatic events [16, 17], and has been shown to have a positive impact on the mental health outcomes of survivors of sexual violence [18]. On the other hand, social support is particularly challenging to build in such populations, as self-isolation and social withdrawal are common consequences of traumatic incidents such as torture and other forms of ill-treatment [19]. Previous studies have shown that social support can be a protective factor for mental health conditions, unlike actively searching for social support [20, 21].

Due to the heterogeneity of understanding and definitions of social support across different contexts, and the general lack of evidence on how social support is perceived, the incorporation of social support in models of care is challenging, particularly in migration contexts with a great cultural diversity. The Greek islands are such a context, where, due to the increase in mixed migration flows over the past years [22], and the high rates of traumatization among those displaced [23, 24], the need to integrate social support into models of care has grown rapidly.

During 2018, a total of 32,497 people arrived on the Greek islands [25]. Since the EU-Turkey statement in March 2016, new arrivals to the island are held there until a number of legal or administrative processes are completed [26]. This process can last several months or years [27], with restricted movement and limited provision of healthcare [28] and social support. Despite the established legal provisions on reception conditions for vulnerable persons,^1^ the lack of sufficient human resources and heavy bureaucratic legal processes result in vulnerable persons living in substandard reception conditions.

A number of authorities and organisations are engaged and responsible for the protection and provision of services for asylum seekers on Lesvos Island. Médecins Sans Frontières have consistently provided medical and mental health services to migrants and asylum seekers contained on Lesvos Island since 2015. However, medical teams have been overwhelmed by the scale of the mental health consequences of this containment, and by the challenges in delivering mental health care when social support is compromised [29, 30].

The importance of social support and its implications for individuals with mental health conditions has previously been studied in resettled populations [9, 10, 31] and amongst torture survivors [32, 33], but the modalities of provision of social support to such traumatized individuals in a setting of containment during the asylum process have not been documented. As the conditions of containment can be anticipated to have a considerable impact both on the needs for social support among traumatized individuals and on the scope and capacity to provide social support, we conducted this study to explore how MSF beneficiaries on Lesvos perceived and defined social support, their experiences of social support and if and how they believed their needs were met.

## Methods

### Study design

This qualitative study was conducted with migrants and asylum seekers enrolled in MSF provided mental health services on Lesvos Island. We utilised sequentially conducted free-listing interviews to identify initial perceptions and definitions around social support, followed by in-depth interviews for comprehensive exploration, upon which the main findings of this paper are based.

### Study period

Interviews were conducted during a 2 week period in August 2018. The free-listing interviews were conducted over a period of 4 days, followed by the in-depth interviews.

### Study site

The study was conducted amongst patients registered at the MSF mental health clinic on Lesvos Island. Migrants and asylum seekers in this setting are accommodated in different camps (Moria and Kara Tepe) and shelters. Actors providing services have varied over time, depending on the funding and coordination mechanisms in place for the asylum seeker response. The vast majority of the participants in the study were accommodated in Moria camp.

Moria, the reception and identification centre and main camp in Lesvos, had an original capacity to host 2,900 people. It increased this capacity to 3,100 people, but as of August 2018 hosted approximately 7,500 inhabitants [34]. This congestion combined with continuous new arrivals has resulted in the decline of reception and living conditions. Legal, medical, psychological and other support is extremely challenging to access in Moria [30].

People arriving on Lesvos undergo an extremely complex administrative procedure. Following the EU-Turkey statement [26], migrants and asylum seekers arriving on the islands are blocked from moving freely to the mainland. Those who are deemed vulnerable can have these restrictions lifted, but identification of vulnerability is slow and the accommodation capacity on the mainland remains limited, resulting in the containment of a large vulnerable population many of whom suffer from trauma incurred before or during their flight.

As of October 2016, MSF increased its activities into a medical package for migrants and asylum seekers living in this containment context and having severe mental health conditions, and/or medical complications linked to torture and sexual violence. MSF works in close collaboration with legal organisations to help people access such services. Considering the important impact of an individual’s socio-legal situation on their mental wellbeing and recovery [35–37], MSF also introduced a component of social worker services into its model of care during April 2017. The role of these social services is to assist with access to legal aid, facilitate accommodation or improve the current accommodation status, facilitate access to secondary services (such as the issuing of a social security number) and the provision of information. Additionally, social workers have been acting as one-to-one advocates for the relocation of vulnerable people to the mainland, ensuring access to care that is not available on the island.

### Study population

Those eligible for the study included male and female adult migrants and asylum seekers who had arrived on Lesvos Island after the implementation of the EU-Turkey statement and who had been admitted to the MSF mental health clinic (situated in Mytilini, the main city of Lesvos, 8 km from Moria camp) for severe mental health conditions, or for conditions related to violence and trauma. The main nationalities in this population at the time of the study were Syrians, Iraqis, Congolese, Afghanis, Iranians and Cameroonians, and the majority of patients in the clinic were male.

Participants eligible for the free-listing interviews were selected by the principal investigator, based on the week’s appointments in the clinic. At the end of each free-listing interview, participants were asked if they would be willing to take part in a further in-depth interview. The final decision on the eligibility of patients for interviews was taken by the MSF Mental Health Activity Manager so as not to include extremely vulnerable patients whose participation in the study could destabilise their treatment. Eight participants were not eligible to participate in the in-depth interviews as determined by the Mental Health Activity Manager, four declined to participate, two did not attend the interview appointment and one was interested to participate, but had insufficient availability to do so. In total, 32 people participated in the study: 31 in the free-listing interviews and 17 in the in-depth interviews, with 16 participating in both.

### Data collection and analysis

In order to explore definitions and perceptions of social support, data were collected in two phases. In the first phase, individual free-listing interviews [38, 39] were conducted whereby individual lists of terms relating to social support were created by each participant, which reflected the most important terms from their perspective [40]. All of the free-listed forms of social support mentioned by participants were noted. We also used a technique known as semantic cueing, which has been reported to increase the length of lists by 40% to assist participants when giving examples for their lists [41].

Social support was introduced to the participants in the following way before beginning the free-listing exercise, so that they were free to identify their own definitions: “Social support helps you endure or overcome difficult situations in your life. Everyone has different experiences from the day he/she was born. During your life span you may support others (being a provider) or be supported by others (being a recipient). What we (people) understand as social support is different because of our different backgrounds”.

During the free-listing, participants were asked to “list all the forms of social support that they know of”. When the first list was completed then the participant was asked to “think of all the kinds of social support that are like “A” (A being item on their initial list)”. This procedure was carried out for all the items on each individual’s initial list.

Once the free-listing interviews had been completed, often-cited terms were grouped according to the similarity of their meaning. Outliers were further explored during the in-depth interviews. In-depth interview guides were initially developed during the study design stage and then revised based on the definitions of social support given during the free-listing exercise. Questions asked during in-depth interviews focussed on perceptions of social support, what kind of support people could access and how, whether they felt their needs were met and what their challenges were and how/if they overcame them.

All in-depth interviews took place in an empty office near the MSF clinic, or near Moria camp if interviewees preferred. Interviews were conducted in English, Farsi, Arabic or French and most were supported by one of three translators. Interviews lasted between 60 and 100 minutes, and we stopped conducting interviews once we reached saturation, in which no new information was emerging. Transcription and translation of audio-recordings were conducted by the translators. Transcripts were coded manually and thematic analysis applied. All interviews were conducted by three investigators (two female and one male), supported by another female co-investigator, and all interviews were accompanied by a translator. Analysis of the data was conducted by the principal investigator and results were discussed and validated within the research team.

### Ethical considerations

Ethics approval was granted by the Médecins Sans Frontières Ethics Review Board, Geneva, Switzerland (study ID 1826) and the National School of Public Health Ethics Committee in Athens, Greece (study ID 892). Verbal informed consent (in English, French, Farsi or Arabic) was obtained from all participants before conducting free-listing interviews and again before conducting in-depth interviews. Verbal consent was considered appropriate in this context due to concerns from many asylum seekers about signing documents, especially as some may link signing documents with previous experiences of torture, such as coercive interrogation methods. None of the members of the research team conducting the interviewers were involved in the provision of care to participants, nor were they working in the project from which interviewees were recruited. We also ensured that potential participants understood that the study was separate from MSF’s routine services and that their decision to participate (or not) would not affect their access to care now or in the future.

### Terms and definitions

The concept of community, which also came up frequently during our study, has been defined as a “space” in which people form a grouping based on common characteristics [42], values [43], interests [44], sense of solidarity [45] or living within the same geographic boundaries [46].

## Results

Among the total 32 participants, we conducted 31 free-listing interviews and 17 in-depth interviews, as shown in Table 1. Participants ranged in age from 18 to 48 years, and more than half originated from Sub-Saharan Africa, reflecting the predominant nationalities of the MSF patient population. 22 participants were male and 10 were female.

**Table 1:** Socio-demographic characteristics of interviewees

#	Free-listing	In-depth interview	Sex	Age range	Country of origin	Language	Family status
1	√	x	male	25–35	Senegal	French	single
2	√	√	male	36–45	DRC	French	married
3	√	x	male	25–35	DRC	French	single
4	√	√	male	36–45	Cameroon	French	married
5	√	√	male	< 25	other West African country	French	single
6	√	√	male	36–45	Cameroon	French	married
7	√	√	female	< 25	Cameroon	French	single
8	√	√	male	25–35	Cameroon	French	married
9	√	x	male	25–35	Nigeria	English	widowed
10	√	x	female	36–45	Cameroon	French	married
11	√	√	female	25–35	Syria	Arabic	married
12	√	√	male	< 25	other West African country	French	single
13	√	√	female	36–45	DRC	French	single
14	√	x	male	25–35	other West African country	English	single
15	√	x	female	25–35	Iraq	Arabic	married
16	√	x	male	< 25	Iraq	Arabic	single
17	√	x	male	< 25	other West African country	French	single
18	√	x	male	< 25	Afghanistan	Farsi	single
19	√	x	male	25–35	Nigeria	English	single
20	√	√	male	25–35	Iraq	Arabic	single
21	√	x	male	< 25	Afghanistan	Farsi	single
22	√	x	female	36–45	DRC	French	separated
23	√	√	female	46–55	Syria	Arabic	widowed
24	√	x	female	< 25	Iraq	Arabic	married
25	√	x	male	25–35	Syria	Arabic	single
26	√	√	male	25–35	Iran	Farsi	married
27	√	√	male	36–45	DRC	French	single
28	√	√	male	36–45	Cameroon	French	single
29	√	x	male	< 25	Iran	English	single
30	√	√	male	< 25	Afghanistan	Farsi	single
31	√	√	female	25–35	Afghanistan	Farsi	separated
32	x	√	female	46–55	Syria	Arabic	widowed

### Phase 1; free-listing interviews

#### Conceptualization and perceptions of social support

**Fig. 1:**
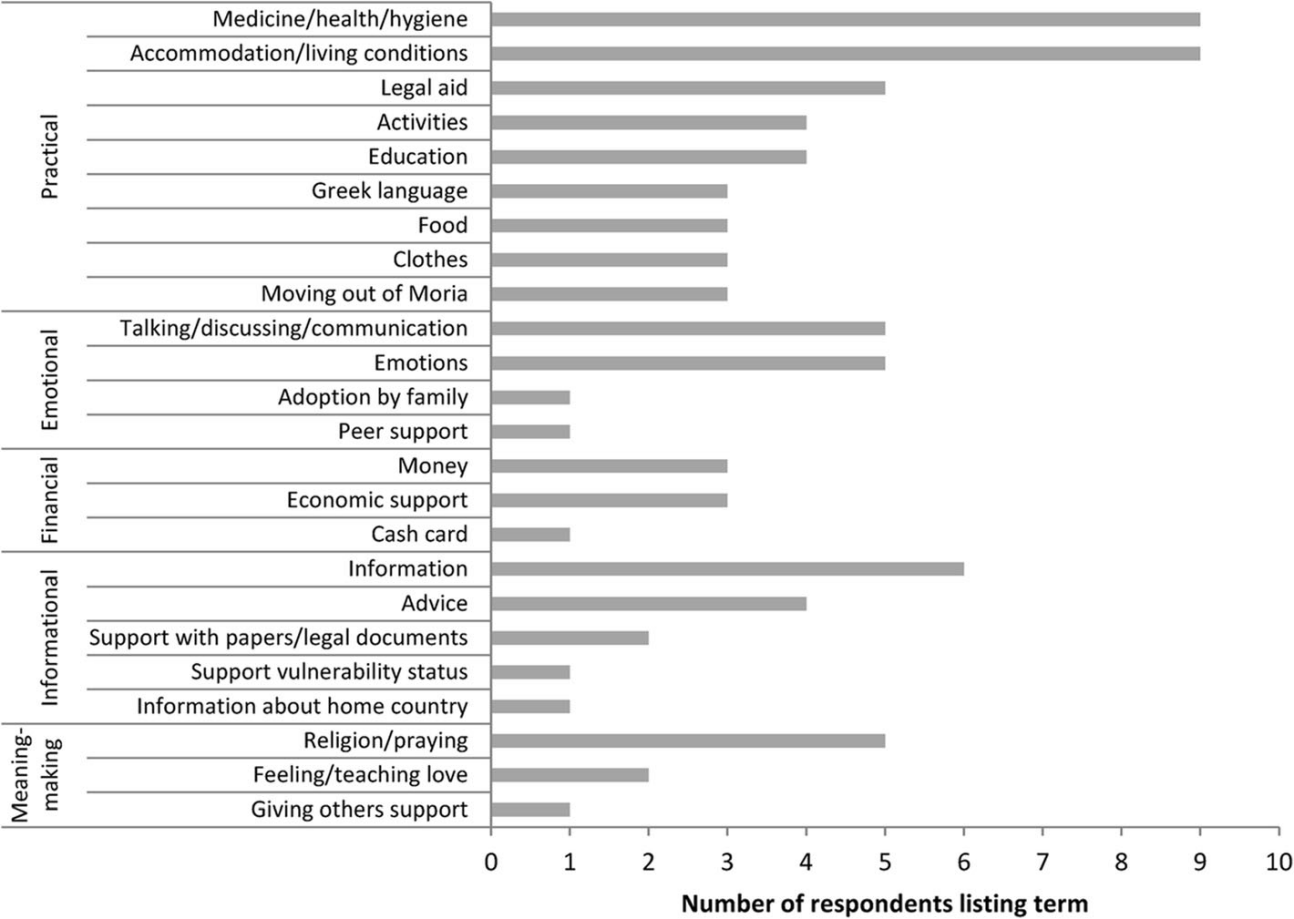
Categorization of forms of social support given during the free-listing interviews

**Fig. 2:**
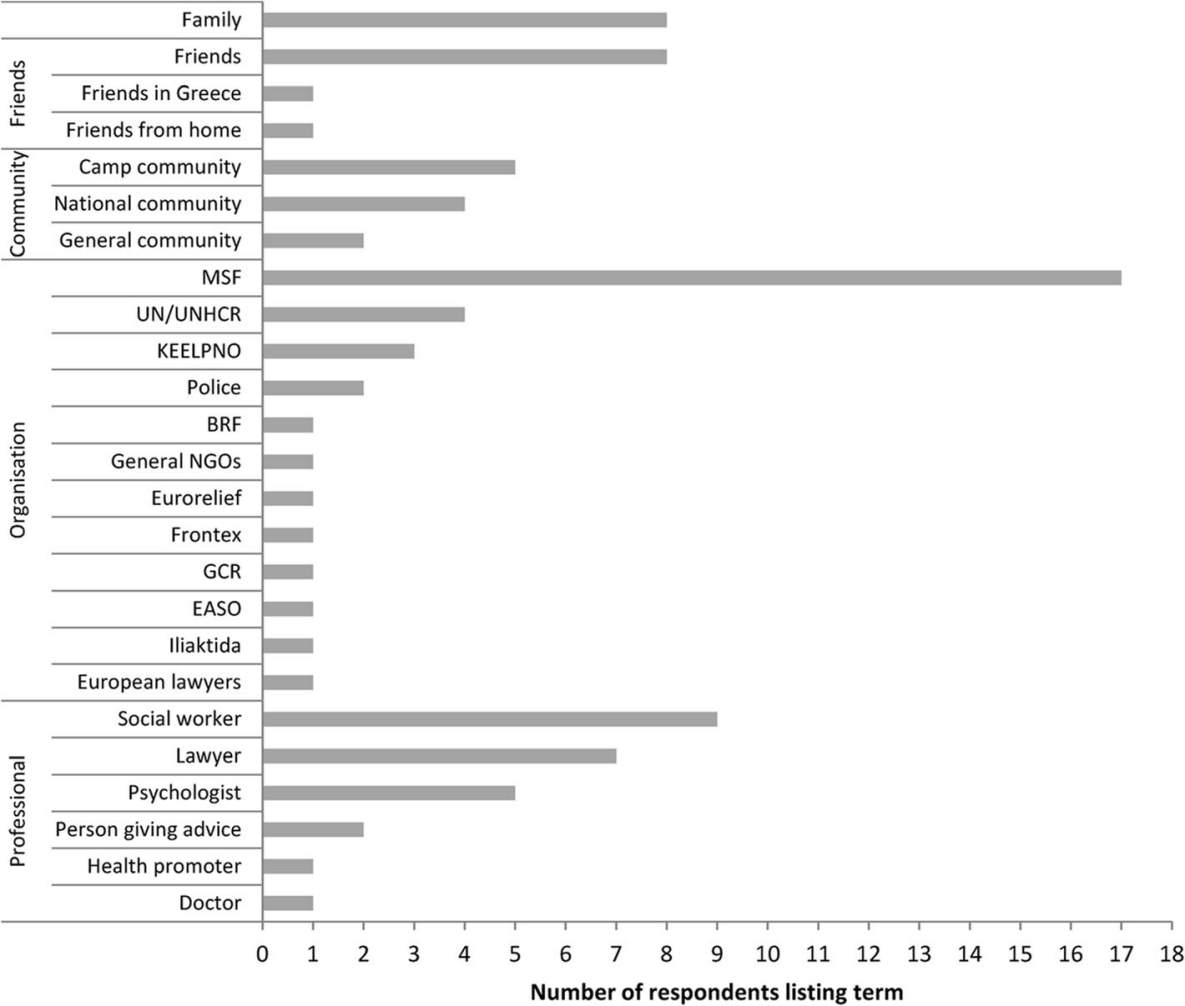
Categorization of people or organisations named during the free-listing interviews

When asked about their thoughts on social support during the free-listing exercise, participants gave examples^2^ of different functions of support that we categorized into practical, emotional, informational, financial support and meaning-making. As shown in Figs. 1 and 2, certain aspects of social support were perceived to be of particular importance for the respondents who described practical support (“accommodation”, “legal aid”, “food”, “medication”); support in the form of information about the asylum procedure, their rights, essential services, or activity opportunities; and emotional support, either formal, through psychologists, social workers, doctors, lawyers and other professionals, or informal, through friends and family members (“communication”, “information”, “guidance”, “advice”).

The participants also underlined the importance of organisations (naming a specific organisation or professional); then friends and family; and lastly community, when describing the structure of a supporting social system. Participants expressed that social support was provided by organisations and professional agencies but was also received through the “*contact with people that are here for long time*”, “*older people in the camp*” or “*women that travelled they together*” and “*a person who can give advice*”. Participants used relational terms to describe social support: “*social support is like mother*”, “*social support is teaching my children the love for life*” and highlighted that it should be “*not only for the unprivileged but for everyone in need of support*”. One participant also emphasised the holistic character of social support by stating that it is “*what improves the living standards of an individual.*” Another participant stated that social support is related to the provision of health-related services, including those offered by NGOs.

### Phase 2; in-depth interviews

The study population composition, in terms of nationality, number of months spent on Lesvos, and marital status, is indicated in Table 2. The majority of interviewees were single men of African origin who had resided in Moria camp between 2 months and 2.5 years. Countries of origin included Syria, Afghanistan, Cameroon, DRC, Iraq, Iran and Mali.

**Table 2:** Socio-demographic characteristics of in-depth interview participants

#	Sex:	Country of origin:	Age range:	Family status:	Status on Lesvos:	Months spent on Lesvos:
P1	Female	Syria	25–35	married	Family	> 6
P2	Female	Afghanistan	25–35	separated	With children	> 6
P3	Female	Cameroon	< 25	single	Alone	6–12
P4	Female	DRC	36–45	married	Alone	6–12
P5	Female	DRC	36–45	single	Alone	13–18
P6	Female	Syria	46–55	widowed	With children	> 6
P7	Male	Cameroon	36–45	married	Alone	6–12
P8	Male	DRC	36–45	married	Alone	6–12
P9	Male	DRC	25–35	married	Alone	13–18
P10	Male	Iran	25–35	married^a^	Alone	6–12
P11	Male	DRC	36–45	separated^a^	Alone	6–12
P12	Male	Afghanistan	< 25	single	Alone	6–12
P13	Male	Cameroon	25–35	single	Alone	< 24
P14	Male	Cameroon	36–45	single^a^	Alone	> 6
P15	Male	Iraq	25–35	single	Alone	6–12
P16	Male	Other West African country	25–35	single	Alone	18–24
P17	Male	Cameroon	25–35	single^a^	Alone	> 6

^a^Has children who are in the country of origin or still in transit (10)

Two main themes emerged from the in-depth interviews: i) Availability and accessibility of services, ii) Personal relationships and informal networks.

#### Availability and accessibility of services

During the free-listing interviews, when asked to define social support, the vast majority of participants described forms of social support that were provided by organisations. These examples were then explored further during the in-depth interviews. Most participants described the formal social support they received as being inadequate. Many of the functions of social support perceived as inadequate, described below, would fall under the category of practical and informational support provided from the different organisations on Lesvos. Participants stressed how the poor quality of services and the barriers accessing these types of formal social support had a negative influence on their lives.

The insufficient quantity of food, long waiting lines and queues were a frequent source of tension and fighting between people staying in the camp:

“The food queue is a big problem for women or people who have small children because you need to go there two hours before [distribution] and wait until the food arrives. [I]t is crowded and normally people who are at the end of queue don’t receive any food so there are always a lot of clashes and fights.”

Participant 2

This participant found other ways to support himself:

“From my experience and what I saw, people go to [name of organisation], for bread and food. [A]nother place with good people [is the one owned by Greeks]. They own a restaurant. Each week they invite people to eat”

Participant 12

Protection and services provided by the police were mentioned as a form of social support by some participants during the free-listing interviews. During the in-depth interviews, many interviewees talked about feeling insecure inside Moria, and there were differing views about the level of protection provided by the police:

“The police are useless here. If the police exist or don’t exist, nothing will change. People are hitting and fighting [each other] in front of them, and the police just keep watching. One time, there was a fight and the police came. They started shouting at us from afar and throwing tear gas and they keep shouting at us until we choked and we were about to die from the coughing.”

Participant 6

“The policemen protect me from everybody; they also protect other people from [other people] that have quarrels there.”

Participant 11

The provision of information was frequently mentioned as a form of support during the free-listing exercise. This was considered to be of great importance to the participants, who discussed the lack of formal information and orientation about available services (including medical and legal assistance), their rights and the procedures. Participants stressed the importance of informal networks as their main source of information. Difficulties in obtaining legal support were particularly emphasized.

“There is no official source where you can go and get information. The official source is the person who gets on well with you. He, at least, can tell you the information that you need. Apart from him, there isn’t anywhere else you can go. The information lies around…”

Participant 14

“At the beginning I didn’t know who to speak to. I didn’t know that I needed a lawyer. Some people told me that there are lawyers here and I visited one of them, it was very difficult. I contacted five lawyers but it was still difficult to have an appointment.”

Participant 12

The provision of medication and healthcare were also seen as part of social support for the participants in the first phase of the study. Here, the access to and availability of medical services within and outside of the camp were discussed. Interviewees found it challenging to access medical services for several reasons, with some choosing to no longer access services and others seeking medical assistance outside of the camp:

“I remember one day I had a toothache, I went to the doctor and he gave me some medicine. I remember one day my tooth swelled. I went to the doctor and he told me that my tooth needed an extraction and I would have to come back the next day. When I went the next day, the guard at the door did not let me enter and told me to go. I did not go there again”

Participant 15

“When people get up to go to the hospital, they have to be out of bed at 4 o’clock in the morning. They take their blankets with them to sleep there until 8 o’clock, the time the doctors arrive. They know as well that at 12:30-13:00 it’s time for food. Everybody has to wait in a line. So when you see the people leaving, you have to follow them. If you miss the food time, you will not eat.”

Participant 17

One interviewee suggested that services should be more visible and accessible to the population:

“More help to the people in the hospital in Moria, maybe some things could be more visible, closer to us to find”

Participant 5

Communication and a lack of a common language was also a challenge when trying to access services:

“I always speak in French at the hospital […] Even in MSF there isn’t anyone that speaks Lingala. But there a lot of Congolese, a lot. There are a lot of black people. There are a lot of black people in Moria… There is just one friend there, who is also black, that speaks French. There are a lot of interpreters, but their French is not the one we speak in our country. It is possible that you say something but the interpreter says something else. As you are a passenger, it’s the first time they see you… So, dignity in the hospital? No…”

Participant 9

“The social assistant is polite but the problem is that we don’t understand each other. She speaks and I don’t understand, I speak and she doesn’t understand.”

Participant 3

Participants talked about the lack of interest they perceived authorities to have in their suffering, with this participant highlighting the importance of being trusted by the service providers:

“All these people who direct Moria should be a little bit more sensitive to suffering because if you don’t take into consideration the suffering of the person you are talking to, if you don’t see it clearly, you don’t live what he is telling you at that moment. You must first of all believe in his words. When you believe in his words, the rest is quite simple. Because if I see that they don’t treat me well, I will explode. For example, last night, somebody told me that if he didn’t leave the next day, he would hang himself. If I say this to the authorities, they will not take it into consideration.”

Participant 14

#### Personal relationships and informal networks

When people described their relationships with others in Moria, they did not talk about “friends” but spoke instead of “*people that they know*” or “*people that they just talk to*”, including their roommates, neighbours or people from the same country of origin. The structure of their networks and whether the members of their networks were providing some sort of emotional or other function of social support were also discussed.

Some interviewees described isolation, with people spending time on their own or turning to prayer and religion – including attending churches within Moria camp - for support:

“I don’t have other friends. So I speak often to God. He listens to me and I can tell everything to him. He has really, really, really my trust. I speak. And he listens.”

Participant 13

“I stay on my own. I spend time on my own to avoid problems. Some people drink beer, for example, and I don’t drink beer. There are people who drink and then they make problems. Someone will say one thing, someone will say something else. I don’t like it so I stay on my own.”

Participant 8

This interviewee described how spending time alone is beneficial:

“All [anyone talks about] is about Moria and the way of living there. I prefer to stay alone sometimes and not with others, because I cannot stand hearing about problems all the time. There is nothing good to say about Moria so it is better not to discuss about Moria. I prefer solitude.”

Participant 12

Interviewees described feeling excluded and stigmatized by their neighbours and the people in the camp with whom they shared accommodation such as ISO boxes (a type of container). At times, this was linked to their mental health problems, which could cause some people to scream during the night or wet their bed. In the examples below, they describe how they felt judged by people who called them ‘crazy’.

“[M]any people treat me like a crazy man. They say that I am a sick person. They say that I have nightmares that I don’t care how I react. They say that I am crazy, that I shout a lot even when I don’t have anything to tell them… Once I went to pray with the people from my community, and they sent me away. They treated me like a crazy person…”

Participant 13

“I don’t know people there. They tell me that I often pee in bed, I often scream during the night, that’s what they say. They also say that I’m crazy, that I’m out of my mind.”

Participant 11

Interviewees often relied on a single person or friend to provide them with emotional support or information about other organizations and services. In some cases, these relationships ended when the person left the island and interviewees were unable to maintain contact with them. Several participants recalled such experiences:

“I discussed with her […] She advised me […] But she left; I am now the longest staying person inside the container. I wanted to leave with the “mama” who was helping me and has left for Athens.”

Participant 3

“In comparison to other people who were chasing me away, she was trying to calm me down. We had a friendly relationship for three months but then she got the decision about her asylum card and she left for Athens.”

Participant 11

“If I have a problem I have one person to talk to, she’s also from Congo. She is someone I met who is in another container in the camp. I met her here.”

Participant 8

“Only my friend helped me to get transferred to a new place. It was my friend that helped me with the cash card. He was the one who took me everywhere and showed me everything. He took me to the hospital. He even told the Cameroonian community that I suffer a lot. He helps me a lot. He gives me a lot of courage. Sometimes when I am feeling desperate, I speak with him. He knows how to make me feel better.”

Participant 7

Interviewees who had their families with them or social networks described some limited “happy” experiences within their daily life in the camp. Examples included being able to talk with their family members, seeing their children going to school and making progress, or communication and daily interactions with family that gave them a sense of belonging, respected them and valued their presence:

“…when they are going to school, I am feel happy with them: when they are playing and when I see them happy, I am happy…my children and my family and my relatives… friends, no. Friends give me nothing except a headache... For private or special problems usually I go to my husband. When I am nervous I go to my husband, and when I am happy or sad I go to my husband.”

Participant 1

Community was not defined homogeneously by the participants: most considered people of the same nationality as their community, whereas others considered a group of people living together or people with other common characteristics as the community to which they belonged.

Participants had differing views about the role community played in offering them support, with some, such as the interviewees cited below, describing positive experiences of the role community played in offering them support:

“There were already a lot of people in the camp when I arrived. So they [people] came and talked to the “new” ones and they explained to us where to get water and food.”

Participant 4

“The Cameroonian community helped me but I never shared my story with them. They collected money, because I didn’t have any, to take me to a psychiatrist and to buy my medication.”

Participant 7

Others described their community as an informal network that allowed the exchange of everyday information, but such exchanges were often felt to be superficial and did not result in real interaction. Some mentioned a lack of solidarity, resulting from everyone being preoccupied with their own problems:

“I’d like to have the power to help people, but I also have my own problems.”

Participant 12

“Each one only cares about him- or herself. Honestly I do not socialise with anybody or visit anybody.”

Participant 6

“We know each other, we greet each other, but nobody participates in clashes and fights to support other people just because he or she is [nationality]. If there is a problem they help each other, but if an [nationality] was being beaten by people from other nationalities they wouldn’t intervene to help this person…”

Participant 10

Interviewees highlighted the cultural differences between people of different nationalities living in the camp, or heterogeneity within people from the same country of origin, which created tensions and did not foster a sense of cohesion:

“Every day we have problems with each other: 27 different families, 27 different social classes, 27 different beliefs and religions. We are very different from each other. We [nationality] we are not like [other nationalities] who are similar to each other. Among the [nationality] here we have people from different economic, cultural and social ranks.”

Participant 10

Some were competing for their basic needs:

“It’s not easy for somebody to give his bottle of water. I mean, you ask somebody for a drop of water and he says to you “Be careful, it’s for the whole day; if I give to you, what I will have then?” That’s not good, not correct. So, even inside a community, it’s not easy to find a person who isn’t selfish…”

Participant 14

Interviewees described creating distance from Moria and the challenges associated with living there by seeking solitude or forming friendships with the local Greek community or volunteers, instead of others living in the camp.

This interviewee described how spending time outside the camp and with volunteers is a positive experience because it allowed them to create distance from the camp:

“When I am outside Moria, I feel that I can live; I feel that after Moria I will have my own life […] But, in Moria we find also people like you [Non-Governmental Organisation staff] who bring everyday joy in our heart. People like you who are working in the camp. Sometimes they [NGO staff] come and take some young people, five or ten of them, and go to eat; that’s nice. They go out together, or they take a walk by the sea and after they drive them back to the camp. They cannot take them or us elsewhere, but at least we can pass our time together, discuss, do things together and change our minds.”

Participant 14

## Discussion

In this study we explored definitions, perceptions and experiences of social support of migrants and asylum seekers contained on Lesvos Island and enrolled in MSF mental health-care services. Social support can be summarised as a way of meeting individual needs through the provision of means and resources in order to manage a specific situation. Social support was perceived to include the provision of shelter, food and health-care, financial support and emotional support, as well as information. Social support can be provided informally through friends, family or community members or formally through organisations and health-care and other professionals.

The main themes emerging from the second phase of this study included challenges that migrants and asylum seekers face on Lesvos Island, including poor service provision, lack of community, and challenges with personal relationships; all amplifying or leading to isolation.

As shown in the results of the free-listing exercise, most participants used examples of support given by other organisations to define the concept of social support. This partly shows the important role of organisations and service providers in this context and reflects the experiences among the individual participants. It is known that individual perceptions about a concept as broad and abstract as social support are influenced by individual experiences [47–49], include different social relationships and interactions [50] or functions of formal and informal networks [51].

We take the results from the free-listing interviews as an expression of participants’ needs. In the in-depth interviews they described the deficiencies in service provision as well as challenges of establishing emotional bonds living in Moria, indirectly showing how those needs remained unmet.

Furthermore, many forms of social support that participants identified during the free-listing are provided almost exclusively by organisations, such as the provision of legal assistance. The interviews revealed the extent to which people in Moria are forced to rely upon other actors for not only their legal needs but food, water, shelter and health care as well.

All of the participants were dissatisfied by the services provided within the camp and in the public health care system, with the main complaints relating to the accessibility of medical consultations and treatment, long queues for food and water and the poor quality of services offered. The lack of service visibility, information and orientation were also challenging, as were linguistic barriers between service providers and the interviewees.

Similar challenges have been described in other settings where lengthy asylum procedures and other factors are associated with worsening health levels [31, 52]. The lack of information and orientation led people to need time to comprehend the complicated legal system and their efforts to understand service provision were further hampered by the lack of support and information from others in the camp. Being contained on the island and having limited options of service provision was problematic and led to frustrations and conflict. This lack of information about the legal system in Greece has been consistent since 2016 [23].

Whilst there are several definitions of the concept of social support [2–4], studies present several aspects of social support that need to be taken into account, such the characteristics of a person’s social network and life, and the kind of support that this network provides, as well as aspects of quality and quantity of such support [6, 7].

Moria camp consists of an extremely heterogeneous group of people who are forced to live together in a containment setting as a result of restrictive policies. People within Moria have different nationalities, unique experiences of migration, various priorities and needs, which are not always met by formal or informal support systems. This sense of living in a ‘forced’ community contradicts the main principles of voluntary community life. This in turn, means that people have very little support from those around them. This was reflected in the social withdrawal of a number of our participants, who preferred to isolate themselves and did want to talk to others or spend time with those around them. Others became reliant upon one person or organisation, which compromised their sense of autonomy and independence, and proved challenging when that single source of support was then discontinued. The poor sense of community cohesion was exacerbated by an antagonistic environment within the camp, with competition for limited resources increasing discrimination between different nationalities and creating tensions between individuals and groups.

The previous traumatic experiences, negative social experiences and low self-esteem, as described elsewhere [53], could be some of the reasons that some participants did not form meaningful relationships with others and in some cases preferred to be alone and isolate themselves.

Additionally, having severe mental health problems meant that many people encountered stigma, exclusion and psychological abuse, which in turn increased their sense of isolation. This can create a vicious circle, as depression has been more closely associated with poor social support than with history of torture [19, 54].

As described earlier, friendships can form in Moria, but such relationships are fragile: because of the geographical restrictions, contact is difficult to maintain once someone has left the island. Those who had managed to develop a support network were in an advantageous position as their daily interactions with friends or volunteers gave them a sense of belonging and a feeling of being valued and respected by others.

Trust was another key issue contributing to a sense of lacking social support within Moria, which has been exemplified in other contexts including the United Kingdom and Ireland where newly arrived refugees and unaccompanied asylum-seeking minors also reported feeling unable to trust anyone [55, 56]. In this study the lack of trust included other people as well as organisations, with participants, for example, losing trust in the medical referral system.

Social networks can take a wide range of forms, and interviewees mentioned their families, NGO staff and other people in Moria as forming part of their networks. As many of the interviewees had arrived in Moria alone, organisations and other people outside of their direct family played an important role in providing information. Informal networks, such as those helping people to learn where to queue for food or how to access a lawyer, typically build and evolve over time, with previous research showing that new arrivals need time to establish themselves within existing networks and systems [57]. External support was thus required to address their daily needs, however this study identified inadequacies in this form of informational support, with a lengthy learning process about where and how to access services in order to receive support. This highlights a paradox of their dependency upon others to introduce them to people who can assist, and the inconsistencies of this informal system to link new arrivals to services.

What was striking in this situation was that people were also reluctant to share information or their limited food or water with others, creating a sense of competition. Increasing the capacity of available services such as accommodation and better systems for food and water distribution would help reduce tensions and the sense of competition for scarce resources. Clear information about services and individuals’ rights, including translation services, should be made available at the entry point, throughout the duration of containment and incorporated in social support strategies that are being implemented.

The sense of isolation and the time people spent alone suggests there is a distinct lack of activities in which people can engage with others, aside from limited educational classes or sports activities offered within the camp. Working towards building such social networks, rather than only directly providing support to people residing in the camp, may be a more sustainable solution for those contained in Moria. Moreover, developing psychosocial engagement activities such as group discussions or counselling groups to build relationships and strengthen interactions should be implemented in order to restore and develop social bonds and possibly decrease the sense of insecurity.

One of the limitations of the study is that as all interviewees were beneficiaries of MSF’s mental health services, they may have been more aware of and dependent upon external support structures than non-MSF patients in Moria, and they may also present more social challenges to create and maintain social interactions. Another limitation is that minors and those with very severe mental illness were excluded from the study as they were considered too vulnerable to participate, thus their voices were not represented. A strength of this study is that social support was firstly conceptualized through free-listing interviews and this information was used to adapt and design in-depth interview guides to explore these concepts in more detail.

## Conclusion

This paper shows the struggles that asylum seekers and migrants with mental illness face during island containment and their difficulties in accessing support from organisations and others around them. Policy makers should take into consideration that the mistrust and loneliness generated by harsh reception and living conditions, the precarious situation linked to peoples’ legal status combined with this unfriendly social environment, the loss of independence across the system and the constant reliance on others are results of EU policies, and political responsibility must be assumed. Actors involved in providing social support are strongly encouraged to engage in activities that work towards building and strengthening peer support networks and communities, rather than providing top-down support.

## Data Availability

The datasets used and/or analysed during the current study are available from the corresponding author on reasonable request.

## Abbreviations

BRF: Boat Refugee Foundation
DRC: Democratic Republic of Congo
EASO: European Asylum Support Office
EU: European Union
GRC: Greek Refugee Council
KEELPNO: Hellenic Center for Disease Control & Prevention
KM: Kilometre
MSF: Médecins Sans Frontières
NGO: Non-Governmental Organisation
UNHCR: United Nations High Commissioner for Refugees

## Footnotes

1 unaccompanied minors; persons who have a disability or suffering from an incurable or serious illness; the elderly; women in pregnancy or having recently given birth; single parents with minor children; victims of torture, rape or other serious forms of psychological, physical or sexual violence or exploitation; persons with a post-traumatic disorder, in particularly survivors and relatives of victims of ship-wrecks; victims of human trafficking (Article 14 [8] L 4375/2016).

2 Terms were listed based on either observations of others, direct personal examples of help experienced, or perceived gaps in provision.
